# Symmetrical and asymmetrical outcomes of leader anger expression: A qualitative study of army personnel

**DOI:** 10.1177/0018726715593350

**Published:** 2016-02

**Authors:** Dirk Lindebaum, Peter J Jordan, Lucy Morris

**Affiliations:** Cardiff Business School, UK, mail@dirklindebaum.eu; Griffith Business School, Australia, peter.jordan@griffith.edu.au; University of Liverpool, UK, lmorris89@outlook.com

**Keywords:** anger, display norms, display rules, followers, leaders, military

## Abstract

Recent studies have highlighted the utility of anger at work, suggesting that anger can have positive outcomes. Using the Dual Threshold Model, we assess the positive and negative consequences of anger expressions at work and focus on the conditions under which expressions of anger crossing the impropriety threshold are perceived as productive or counterproductive by observers or targets of that anger. To explore this phenomenon, we conducted a phenomenological study (*n* = 20) to probe the lived experiences of followers (as observers and targets) associated with anger expressions by military leaders. The nature of task (e.g. the display rules prescribed for combat situations) emerged as one condition under which the crossing of the impropriety threshold leads to positive outcomes of anger expressions. Our data reveal tensions between emotional display rules and emotional display norms in the military, thereby fostering paradoxical attitudes toward anger expression and its consequences among followers. Within this paradoxical space, anger expressions have both positive (asymmetrical) and negative (symmetrical) consequences. We place our findings in the context of the Dual Threshold Model, discuss the practical implications of our research and offer avenues for future studies.

We need these more angry men to bring the equilibrium back a bit. (Follower 3, Major)

In this article, we examine expressions of anger at work from an observer and a target’s perspective to better understand the multi-faceted ways in which anger expressions are perceived at work, and the impact this has upon observers and targets of anger. Our research has particular relevance for the study of leader–follower relationships, for leaders work with and succeed through the behaviors of followers in accomplishing organizational goals (Northouse, 2010). A central element to leaders exercising their influence over followers is the maintenance of respect for the leader by the follower (Bono and Judge, 2004). Therefore, follower reactions to leaders expressing emotions (including – but not limited to – anger) at work have strong bearings on whether organizational goals are accomplished or not (Dasborough et al., 2009; Van Kleef, 2014). After all, leadership is a social influence process (Parry, 1998), and leaders use emotions to direct followers in ways they desire. As the introductory quote suggests, sometimes this can entail the use of anger to meet the demands of a specific task or situation.

As mentioned, we focus upon anger as a key emotion. Anger typically emerges when an individual perceives that they have been treated unfairly or unjustly (Solomon, 1993). In social relationships, anger indicates that something is wrong and produces a concomitant desire for corrective action (Gibson and Callister, 2010). We have selected anger primarily because of its pejorative use in the context of leadership studies, albeit this use is visible in other management domains as well (Bodenhausen et al., 1994; Booth and Mann, 2005; Skjørshammer, 2003). The use of anger in a negative way creates an atmosphere in which our theorizing around anger is increasingly uni-dimensional (see e.g. Berkowitz, 1994; Jordan and Lindebaum, in press), and predominately linked with undesirable outcomes at work. Indeed, anger in leadership studies has been linked to perceptions of un-inspirational leadership (Waldman et al., 2011), petty leader tyranny (Kant et al., 2013) or observer perceptions of reduced leader effectiveness (Lewis, 2000). These studies fall into the category of what Lindebaum and Jordan (2014) recently described as symmetrical assumptions in relation to negatively or positively valenced emotions and their association with negative or positive outcomes at work, respectively. While agreeing that the exploration of symmetrical associations has contributed much to the field, Lindebaum and Jordan (2014) argue that vis-a-vis the presence of certain boundary conditions (e.g. organizational context or tasks), there is under-explored scope to study what they term ‘asymmetrical relationships’. In other words, are there conditions under which, for instance, anger (a negatively valenced emotion) can lead to desirable outcomes at work? Indeed, a small number of studies suggest that anger can have asymmetrical outcomes to produce positive outcomes depending on the situation. Relevant examples can be found in studies on negotiation (Van Kleef et al., 2004), organizational commitment (Stickney and Geddes, 2014), conflict situations at work (Geddes and Stickney, 2011) or self-perceived effectiveness of construction project managers (Lindebaum and Fielden, 2011).

Theoretically, our study takes primary inspiration from Geddes and Callister’s (2007) Dual Threshold Model (DTM) in order to expand further on the notion of asymmetrical relationships between anger and positive or desirable outcomes at work. The model is particularly suitable in the context of this study because it helps explain how anger can have utility in organizations. Outlining the DTM, Geddes and Callister (2007) propose that anger at work can either be suppressed (i.e. not crossing the expression threshold), expressed between the expression threshold and impropriety threshold (i.e. an intensity of emotional display considered appropriate by others), or expressed beyond the impropriety threshold (i.e. an intensity of emotional display considered *in*appropriate and seen as deviant anger by others). The clear message for Geddes and Callister (2007) is that productive outcomes of anger are more likely to emerge when the expression of anger falls between the expression and impropriety thresholds.

Based upon the preceding sections, our aim in this study is to qualitatively examine the notion of an impropriety threshold. Specifically, we explore the impact on followers (as observers and/or targets) when leader anger expressions exceed the impropriety threshold, and to assess the positive and negatives outcomes that this might produce at work. The basic research question we wish to explore is this: *what conditions contribute to the expression of anger that crosses the impropriety threshold being seen as productive or counterproductive by observers or targets of that anger?* While Geddes and Callister (2007: 733) maintain that ‘more negative than positive outcomes will emerge’ as a result of anger displays exceeding the impropriety threshold (i.e. deviant anger), elsewhere (see Geddes and Stickney, 2011) evidence has emerged that inappropriate displays of anger can lead to coworkers and management responding in supportive ways. In our research, we contribute to the theoretical development of the framework of the DTM by establishing a number of boundary conditions under which anger can contribute to producing both symmetrical and asymmetrical outcomes.

Our research has also important implications for leadership practice in terms of understanding a leader’s expressions of anger toward their followers and the impact it has on followers’ views of the leader and the followers’ subsequent behavior. We contend that anger will not always have a negative outcome. Research demonstrates that negative emotion can produce better performance in teams (Jordan et al., 2006; Mitchell et al., 2014), and has been linked to leadership effectiveness in some contexts (Lindebaum and Fielden, 2011).

Our article is structured along these lines. First, we briefly discuss the guiding theoretical emotion-related frameworks of this study. Second, we outline the role of display rules and display norms in the context of work more generally, and in the military context more specifically. We then report findings of our phenomenological study. Lastly, we discuss our findings in relation to the research question and theoretical frameworks used, appreciate its limitations and offer avenues for future research.

## Frameworks for examining emotions in organizations

To assist in interpreting a follower’s perception of leaders expressing anger, we draw upon two major frameworks: the DTM perspective already mentioned and Emotions as Social Information (EASI, see e.g. Van Kleef, 2014). While each provides an insight into expressions of anger at work and their consequences, they share some conceptual overlap in terms of display rules and norms. Diefendorff et al. (2011) note that display rules (i.e. organizationally prescribed rules for the expression of emotion) can emerge – when accepted by the group – as shared norms (i.e. display norms for the expression of emotion) within workgroups, thereby helping the group to determine what are appropriate emotional expressions at work. We argue that display norms may also vary from display rules if the work group accepts that these norms are consistent with the context they work within and consistent with the group history that contributes to that context. These emotional display norms are, therefore, also relevant in relation to applying the DTM (Geddes and Callister, 2007) to our data. In particular, while norms infer a standard of emotional expression, the DTM notes that there are expression and impropriety thresholds that these norms work within.

The final approach we draw on is the EASI model as it provides a link between display norms and how individuals (here more specifically followers) interpret those display norms. Van Kleef (2014) argues that emotional expressions influence the targets of those emotions (and others around them) by triggering a series of mental processes in which the individual tries to make sense of the emotions being expressed. Specifically, Van Kleef (2009, 2014) notes that both the observer’s affective reaction (e.g. emotional contagion, reduced liking or reciprocal anger) and the inferences (based upon cognitive appraisal of the situation) concerning the emotion directed toward the individual drives her/his eventual behavior. Key here is the level of motivation the observer develops to get a deeper understanding of the situation at hand (Van Kleef, 2009). This is particularly significant when considering the discrete emotion of anger. Whereas other negative emotions such as sadness and fear generally produce avoidance responses, anger is one of the few negatively valenced emotions to produce an approach response pattern (Marsh et al., 2005). Further to this, Van Kleef (2014) argues that the amount of effort the observer puts into understanding an emotion will be dependent on their motivation and the context in which the initial emotions were expressed. Clearly, a part of the context for employees is the display norms established by the organization. In our study, we explore what role context plays in the expression of anger and how followers responded to those anger expressions and their motivations for responding in specific ways.

## Display norms in organizations and the military

As noted earlier, the focus of our research is on the observer’s and target’s perceptions of anger expressions in light of the display norms established by the organization. Seen in this light, norm content is concerned with where, why, how and to whom anger is expressed (Gibson and Callister, 2010). Norm strength, by contrast, determines the degree to which members agree with these norms, and also what the repercussions of breaking norms will be (Gibson and Callister, 2010). In light of these multiple considerations, Diefendorff and Greguras’s (2009) work on norms shows that display norms and rules in the workplace can be incredibly complex and nuanced, making them a challenging construct to study.

The cultivation of anger and aggression (as influenced by display norms and rules) has been discussed in wider organizational contexts. For instance, Rafaeli and Sutton found that bill collectors were expected to display negative emotions to fuel a certain amount of aggression toward individuals in an attempt to collect debts (Rafaeli and Sutton, 1987; Sutton, 1991). Still, Diefendorff and Greguras (2009) argue that many organizational display norms encourage no expressions of anger, or at least expressions of lesser intensity than it is really felt and that control of anger is important. Gibson and Callister (2010) note this paradox and argue that examining follower perceptions of display rules and norms at work with regard to anger, and how this impacts their reactions to certain emotional expressions, remains to be understood. We note that the DTM, which places emphasis upon the norms that shape the impropriety threshold, provides a framework to increase our understanding of this phenomenon.

Moving on to considering anger display norms in the military, there is a public perception of military personnel experiencing significant anger and aggression both in training and in their working day. Linkh and Sonnek (2003) assert that the army’s attitude to anger is somewhat paradoxical. On the one hand, it seeks to cultivate anger and aggression in soldiers in training situations to prepare them for combat (e.g. staff sergeant yelling at soldiers). On the other hand, the military also places a high value on self-control and conformity (both reflect that there are expectations around emotional display norms). We note, however, that anger and aggression are not the same (see Geddes and Stickney, 2011), albeit they can be used in the military context sometimes simultaneously to achieve desired outcomes. As we have discussed, anger is a reaction to perceptions of injustice, whereas aggression is an instrumental response where an individual seeks to dominate others (Pinto, 2014). On this basis, individuals can be angry and aggressive, angry and not aggressive, or not angry but aggressive. This distinction is not always clear.

Discussions of anger within the military predominantly consider it a negatively valenced emotion, and associate it with violence (Linkh and Sonnek, 2003; Taft et al., 2007) and Post Traumatic Stress Disorder (or PTSD – Morland et al., 2012; Teten et al., 2010; Worthen, 2011; Wright and Russell, 2013). While this should not lead to the conclusion that anger in the military is always caused by PTSD, coupled with negative outcomes, it is a significant example of the inherent assumption and treatment of anger as an undesirable emotion. This notion is similarly reflected in the leadership literature (Madera and Smith, 2009; Waldman et al., 2011). Taken together, there are often competing perceptions on the usefulness and utility of anger expressions in the context of the military. It is exploring these competing perceptions that renders our study theoretically (in the context of the DTM) and practically relevant.

## Leader expressions of anger and their perceived consequences

The examination of anger in leadership studies has generally supported the symmetrical assumption of a link between anger and negative outcomes. For example, Lewis (2000) notes that leader anger expressions tend to reduce observer perceptions of leader effectiveness. Similarly, Madera and Smith (2009) found that particularly in times of crisis, leaders displaying anger were evaluated less favorably than those expressing sadness in response to a failed product. Goleman (1998) also suggests that anger expressed by leaders is related to leader ineffectiveness, as it signifies a lack of emotional control. Perhaps as a result of this common perception, it has been reported that employees (including leaders) are inclined to hide or suppress feelings of anger owing to a concern for maintaining professionalism, as well as a fear of sanctions if the anger expression is perceived as violating display norms (Geddes and Stickney, 2011; Kramer and Hess, 2002; Stickney and Geddes, 2004).

Overall, these findings represent a symmetrical assumption between leader anger as a negatively valenced emotion, and negative consequences as perceived by others, even though ‘organizational life does not represent such a neat juxtaposition’ (Lindebaum and Jordan, 2012: 1027). While comparatively little research has examined conditions under which anger expressions can have positive consequences, there are some notable studies where leader anger expressions have led to positive consequences for the individual and/or the organization. For example, Tiedens (2001) found that people confer more status to those who express anger than those who expressed other ‘negative’ emotions such as sadness. Similarly, diverging from Lewis’s (2000) findings, Lindebaum and Fielden (2011) found that anger expressions in the construction industry were linked to perceived leader effectiveness. Moreover, Van Kleef and colleagues (2009) demonstrated that after a leader displayed anger, followers developed a deeper desire to understand the situation that precipitated the leader’s anger in order to improve the follower’s performance. In a similar vein, studies in the realm of negotiation show that expressions of anger typically aided in extracting concessions from negotiation counterparts, the exception being when anger expressions were perceived as inappropriate by the counterpart owing to a violation of an explicit display rule (Van Kleef and Côté, 2007). Similarly, when the counterpart had a powerful position, expressions of anger backfired by evoking competitive and retaliatory responses. Further to this, a recent empirical study shows that expressed anger predicts perceived improvement with problematic situations at work, while suppressed anger induced perceptions that the situation at work had deteriorated (Stickney and Geddes, 2014). Clearly, the literature presents contradictions regarding anger’s ability to elicit positive *and* negative outcomes, with findings being varied and, therefore, heavily dependent upon the context and the unique situation.

To make better sense of these contradictions, we developed this study examining anger expressions in the military. As mentioned earlier, we draw upon a combination of the DTM (Geddes and Callister, 2007) and Van Kleef’s EASI model (2014) to help interpret our data. Specifically, we employ these theoretical frameworks to assess the perceived appropriateness of leader anger displays as a key contingency to the observer’s processes of affective reactions or inferences. As Van Kleef highlights, affective reactions or inferences can lead to different outcomes in the case of anger expressions, and it is this difference that helps better understand the contradictions identified above in terms of anger expressions and their outcomes at work.

## Method

Consistent with this study’s aim, we employ a phenomenological approach. This enables us to embrace ‘the complexity of the human individual and their actions’ (Ardley, 2011: 637), as well as prioritizing the individual’s own interpretation of a situation in relation to the research question of this study. More specifically, a phenomenological research approach focuses on the subjects’ own interpretation of a situation or experience, their lived experience (Sanders, 1982) and their construction of reality through narration (Groenewald, 2004). It is for this reason that, in describing this study, we follow the traditions of the method and exclude concerns for validity, reliability, objectivity, generalizability and verification (Amis and Silk, 2008). Instead, and consistent with writings of other qualitative researchers (Lincoln and Guba, 1985; Lindebaum, 2015), we focus upon several ‘quality’ criteria of qualitative research. First, we seek credibility instead of internal validity. This implies an authentic representation of the lived experiences of followers. We achieved this in our study by allowing individuals space to express themselves freely with regard to the phenomena under investigation, in addition to seeking respondent feedback from soldiers. Second, we focus on transferability instead of external validity. This involves drawing out the applicability of findings, for instance, in other contexts. As suggested later, there is a nexus between the findings of this study and other studies interested in how the nature of tasks can moderate the link between leaders expressing emotions and follower outcomes (Visser et al., 2013). Third, we consider dependability instead of reliability. Essentially, this factor asks researchers to minimize researcher idiosyncrasies. We have addressed this by way of ‘bracketing’, which involves noting down as many conscious presuppositions as the researcher can establish prior to analyzing the data in order to ensure these presuppositions do not cloud the interpretation of the data (for a detailed treatment on ‘bracketing’, see Holt and Sandberg, 2011; Hycner, 1985; Sandberg, 2000). Fourth, and finally, good research in this paradigm relies on confirmability instead of objectivity. Involved here is a sense of researcher self-criticism, such as changing the scope and labels of the themes presented later in response to discussions among two of the researchers of this study. In addition, this has been addressed by being rigorous in the analysis of data through a systematic reading and re-reading of the interview scripts – as opposed to the creation of researcher distance and non-involvement (Etherington, 2004).

As noted earlier, display norms in the workplace can be incredibly complex and nuanced, making them a challenging construct to study. Therefore, Gibson and Callister (2010) encourage researchers examining display norms in organizations to employ a range of qualitative methods that allow for ‘rich’ descriptions of perceptions of display norms. The phenomenological interview uses minimal structure, allowing both the individual to present their meaning structures as spontaneously as possible, and scholars to investigate the human experience from the inside of the individual using introspection (Osborne, 1994). This was crucial for our study as our research question involved examining the internal processes through which followers perceive leader anger. Readers wishing to immerse themselves further into phenomenology as a vehicle of analysis can consult several existing reviews (Holt and Sandberg, 2011; Sanders, 1982).

### Sample

We have identified the UK military as a relevant organization within which to conduct this study. Military organizations have a clear hierarchical structure with strict lines of responsibility and acquiescence to authority. Thus, the organization operates within a framework where following orders and conformity to the directions of superiors is a norm. As such, it provides an interesting boundary condition for examining anger in the workplace. Selecting a military sample enables us to undertake purposive sampling. This sampling method is essentially strategic and implies an effort to establish a good correspondence between research question and sampling (Bryman, 2004).

A sample of 20 male followers (i.e. soldiers) from a single Infantry Battalion based in the UK was interviewed by the third author, with age ranging from 27 to 42 years. The number of interviews was determined by the data reaching saturation point at that stage, which conforms with other such research (Glaser and Strauss, 1967). Participants were recruited from a cluster of ranks within the UK infantry (i.e. between Sergeant and Lieutenant Colonel). Lower ranks (e.g. Guardsman etc.) were excluded from the sample given that they may lack experience in the field and the ability to suitably reflect upon their limited experiences. Higher ranks were primarily excluded as they have few if any leaders managing their activities and emotions and second, they represent an increasingly small proportion of army personnel.^1^ Explaining this in terms of positions in industry, in selecting the sample, we focused on supervisors and middle management, rather than employees or CEOs. Although it should be noted that given the hierarchy, the followers interviewed are likely to be leaders for other lower-level soldiers too, it is outside the scope of this article to empirically address this circumstance as well. Initially, we used a purposive sample of a range of ranks to build up a broad view of the phenomenon and then snowballing to achieve saturation, as explained above.

### Procedure

Interviews were conducted on site at the participants’ place of work. Prior to the interview, participants were provided with information detailing the rationale of the study, and were then given the opportunity to sign a participant consent form. The questions were open-ended and allowed soldiers to discuss issues in a manner that was meaningful to them. The questions asked allowed the researcher to enter the followers’ interpreted world through their narrative (Groenewald, 2004). As such, Englander (2012) suggests that during phenomenological interviews, it is useful to ask the participants to narrate a situation when they experienced the phenomena under study. Therefore, we decided to include questions such as ‘can you describe a situation when…’ or ‘can you tell me about a time when…’, as a means of uncovering the perceived meaning of the phenomena (Englander, 2012). The focus was upon participants explaining their experiences as being either the target of the leader anger expression, or as being an observer of leader anger directed at another follower.

We followed guidelines for phenomenological interview analysis as recommended by Hycner (1985). Overall, this enables the creation of stronger findings since a ‘clear methodological process is used’ (Gephart, 2004: 458). The first step, according to Hycner (1985), involved transcription of the interview audio file, including the noting down of non-verbal communication. Interviews and accompanying notes were transcribed after every second or third interview. The second step involves what is called ‘bracketing’ in phenomenological analyses. That is, it means ‘suspending . . . as much as possible the researcher’s meanings and interpretations’ (Hycner, 1985: 281). During the interviews the third author actively engaged in bracketing and allowed the interviews to unfold without interpretation. The third step involved listening to the interviews several times to obtain a sense of the whole. In doing so, general impressions and specific issues of the interview were noted down to get a sense of the general meaning of each interview (corresponding to step 4 of Hycner’s guidelines). Once these units of general meaning were distilled, the third author was able to distil the essence of meanings that were true to the data. For example, a unit of general meaning was the claim by some soldiers that the army did not tolerate expressions of anger, or conversely, the perception that anger was required in training situations to simulate the ‘real thing’. Once general units of meaning had been identified in step 5, the second author considered these in relation to the research question to ascertain if they could be used to illuminate it. The next step (step 6) involved including another coder to independently verify units of relevant meaning. Thus, the first author examined the coding of the data to verify the units of relevant meaning. By way of iteratively cross-checking among both coders, themes were modified where necessary (see also Sandberg, 2000), and redundancies avoided. In the next step, we were able to cluster together these units of relevant meaning, where commonalities could be identified. From these clusters of relevant meaning, themes were deduced after three iterations of data analysis. For example, the two units of relevant meaning identified above could be clustered together under a theme highlighting the paradoxical attitude toward leader anger expression. As a final step, the third author re-visited participants in order to validate our interpretations and themes by way of respondent feedback (Bryman, 2004). Participants agreed that these interpretations and themes were consistent with the face-to-face conversations and, more importantly, felt that their experiences were faithfully represented. Below we discuss our findings.

## Findings

The findings represent the key themes determined from the clusters of relevant meaning following specific guidelines for phenomenological interview analysis, as detailed above. We emphasize that, although this inquiry was guided by an overarching research question, it is the nature of qualitative research that interviewees provide the structure of the data, rather than researchers imposing pre-defined structure as commonly occurs in quantitative research (Dey, 1993). Therefore, the data are broader than what we sought to elicit with the research question.

Themes extracted are labelled (i) paradoxical attitudes to anger expression and changing display norms, (ii) symmetrical outcomes of leader anger expression and (iii) asymmetrical outcomes of leader anger expression. Table 1 provides a summary of our findings.

**Table 1.** Summary of findings.

Theme	Sense-making	Implications for understanding link between display norms, affective reactions/inference and outcomes	Examples
**Paradoxical attitudes to anger expression**	Military establishment discourages expressions of anger in recent years; does not want to be seen as autocratic, but rather encourage transformational leadership styles or ‘lovey-dovey’ approaches.Conversely, in practice soldiers still express anger at work as it can be useful in getting points of view across and bringing attention to issues that need to be addressed.	Display norms in the military are changing and thus somewhat paradoxical; expressions of anger still commonly seen at work, however much the military at large tries to combat it.Expressions of anger could be construed as bullying and involve formal punishments, in line with the organization’s established protocol for dealing with bullying.	‘*I think there is definitely a perception that the army is trying to combat . . . the army is investing a huge amount of time with its instructors at all levels to move away from that sort of transactional leadership . . . it’s moving to a far more transformational style . . .’* (Follower 8, Lieutenant)
			‘*If someone was in their office shouting and screaming at somebody . . . no one would really take any notice of it . . . No one really bothers what it’s about, because they know it’s probably warranted*’. (Follower 18, WO Class 2)
**Symmetrical outcomes to leader anger**	Followers had negative emotional reactions to leaders when it was considered unwarranted.Followers observing leader anger toward a subordinate often think the leader has poor leadership qualities.	Both affective reactions and inferences can result in negative outcomes to leader anger. This occurs when leaders transgress display norms. Particularly where observers perceive leader anger as an injustice, they make negative inferences about them, resulting in avoidance behaviors from the target.	‘*People will walk away just calling you names behind your back, thinking you’re an absolute nob . . . People won’t approach you*’. (Follower 12, Captain)‘*He treated* [an individual] *with utter contempt . . . it was a real angry outburst . . . I thought it was horrendous and utterly inappropriate*’. (Follower 13, Captain)
**Asymmetrical outcomes to anger**	Followers respond to leader anger by assessing their performance/behavior and made efforts to rectify the issue and address the anger-eliciting event.When in intense training practices, or out in combat situations, followers respond to leader anger immediately, by carrying out the task required, or mirroring the leader emotion toward the threat.	Where leader anger is perceived as justified, soldiers accept it and make inferences about their performance and/or behavior. Subsequently, they improve their performance behavior.In high-pressure situations, followers respond to leader anger with immediate affective reactions. This involves mirroring the leader’s anger in order to produce the kind of controlled aggression to deal with arduous and stressful situations. Also, their immediate behavioral reactions seem to take place without any time for high levels of information processing or inferences.	‘*He got really angry on the radio. He said “get that fucking cigarette out of your mouth now”. [Laughs] . . . I did it because I realized I had forgotten where I was and forgotten what I was doing. He was right . . . I sorted it immediately and nothing more was said*’. (Follower 19, Sergeant)‘*If you’re on exercise and you’re doing a platoon attack . . . That can result in a lot of . . . shouting; you don’t want to be quietly tapping people on the shoulder and saying, come on, you could possibly do this a bit better. You need to get the point across immediately*’. (Follower 7, Captain)

### Paradoxical attitudes to anger expression and changing display norms

A very common theme found from the interviews was the perception that the UK military’s attitude to anger is changing, manifesting itself in paradoxical attitudes toward anger expression. Many followers reported that anger expression was currently discouraged by the army, or seen as a negative emotion to be suppressed. This was put down to anger being associated with soldier bullying in the 1980s and 1990s, with little sign of improvement according to recent media reports (Townsend, 2014). In consequence, anger expressions have become synonymous with aggression and physical violence for our respondents. Follower 8 (Lieutenant) summed up the reasons for this change in attitude, but also identified potential problems with the new approach:

I think there is definitely a perception that the army is trying to combat . . . the army is investing a huge amount of time with its instructors at all levels to move away from that sort of transactional leadership . . . it’s moving to a far more transformational style . . . There is always the argument that, certainly the instructors, they don’t really believe that in the infantry there is that sort of place for that kind of transformational leadership.

Follower 12 (Captain) also recognized that change was taking place in the military in response to the wider expectations of society:

. . . it’s interesting to see how the army has already changed, and how they are continuing to change it . . . especially now there’s much more of an approachable non-bullying atmosphere, because that is the way society is going . . . the army can’t be seen as being bullying, if they have a ‘brand’ of being bullies, that would be seen as awful.

Follower 3 (Major), however, expressed his concern that the army was forcing soldiers to go too far the other way in their expressions (or lack of expression) of anger:

. . . I think anger and bullying are a murky area that people are nervous about. I think that’s really sad, because I think in the profession that we are in, is such that we need to create mentally tough, physically tough men, who are prepared to go and face adversity. If you can’t take a little bit of anger . . . I’m not sure we are preparing soldiers as well as we could if we were more willing to show our anger . . . Possibly we’ve drifted a bit too far toward the lovey-dovey end of the spectrum, and we need these more angry men to bring the equilibrium back a bit.

The changing attitude of the establishment toward anger expression seemed to account for the somewhat contradictory narratives. For example, Follower 5 (Color Sergeant) suggested that, in terms of anger expressions at work, that ‘*it’s not tolerated*’. Yet, Follower 4 (Sergeant) reported that expressing anger at work was ‘*just the army way*’. Followers seemed to imply that expressions of anger were both simultaneously discouraged and accepted. Follower 18 (WO Class 2) also expressed this paradox by informing the researcher that he believed that talking to someone calmly was more productive than shouting and getting angry. Yet, he went on to say:

. . . if someone was in their office shouting and screaming at somebody, and the company commander walked past, no one would really take any notice of it, it’s just something that has to happen from time to time. No one really bothers what it’s about, because they know it’s probably warranted if it’s happening.

Taken together, these statements underscore the paradoxical narratives that followers produced when asked about the experiences of anger expression in the context of the military. In relation to the impropriety threshold, these findings also highlight – consistent with the DTM framework – that this threshold is not fixed or static; rather, its position is negotiated within a web of individuals whose interpretations of the impropriety threshold can differ greatly.

### Symmetrical outcomes of leader anger expression

There were discernible affective reactions from observers to leader expressions of anger resulting in avoidance behaviors and reflecting symmetrical outcomes. The symmetrical outcomes we found were primarily in relation to the follower’s negative view and reduced liking of the leader who was being angry. Follower 2 (Captain) appreciated chronically angry leaders less, wondering ‘*. . . why are you being a dick all the time!?*’. Similarly, where a leader expressed anger perceived as inappropriate, Follower 12 (Captain) explained:

. . . if someone’s been told to pick litter up round the building, and it’s not been done correctly, to then get everyone back in and start giving them press-ups, and start ranting and raving and swearing at them because they’ve missed a crisp packet . . . It’s a total waste of time because people will walk away just calling you names behind your back, thinking you’re an absolute nob . . . People won’t approach you, you’ll be unapproachable.

Follower 12 exemplifies an observer’s affective reaction to leader anger, and subsequently highlights the propensity for individuals to engage in avoidance behaviors. Similarly, when asked whether they had ever been a target of leader anger, Follower 15 (Sergeant) replied:

I’ve had that done to me actually. The bloke was a bit of a prick. It was nothing, it was absolutely nothing, and he just exploded in front of an office full of people, and I was just like, where on earth did that come from?

Their description of the leader as ‘*a bit of a prick*’ illustrates a predominantly affective reaction to that anger, as it expresses reduced liking for the expresser (i.e. the leader). Follower 15 explained that he put in a formal complaint about that leader’s behavior. Where affective reactions occurred (such as reduced liking for the angry individual or the arousal of negative emotions – consistent with Van Kleef’s EASI model 2014), this was often because the cause of anger expressions was perceived as so slight as to not warrant an angry outburst. Thus, observers and targets felt that expressions of anger were sometimes unfair and consequently this reduced their liking and respect for that leader.

Interestingly, it was not just affective reactions from observers that resulted in symmetrical outcomes. In some cases, followers also drew inferences from leader expressions of anger, to the effect that those individuals were not necessarily seen as ‘good’ leaders owing to their inability to control their emotions. This was usually the case where observers perceived that there had been a serious breach of display norms and what they considered appropriate professional conduct. Follower 13 (Captain) provided an account of this:

. . . when we were in Afghanistan in 2010 . . . there was an officer who was a very charming bloke socially, but, in my opinion, wasn’t suitable for command the level he was at . . . He treated [another individual] with utter contempt, and used to lose his temper with him quite frequently . . . it was a real angry outburst . . . I thought it was horrendous and utterly inappropriate . . . even someone who is really competent, if they are angry all the time, I think less of them . . . I thought, why should I work with you? . . . if you’re always angry, you quite clearly can’t control your emotions; what happens in a difficult, stressful situation? You’re going to completely lose it.

The thread of thought here illustrates the follower’s concern over the leader’s ability to lead and manage his emotions. Subsequently, the follower expressed a doubtful ‘*why should I work with you?*’. Importantly, the insight into the follower’s thought processes exemplifies the negative inferences he draws from the leader’s anger expression. Follower 13 (Captain) particularly took issue with the fact that the angry leader had singled out the target of his anger repeatedly. This echoes an account by Follower 7 (Captain) on leader anger, particularly that:

. . . the army will tolerate a bollocking from time to time in the form of a verbal dressing down; it will not tolerate more than that, especially if its discriminatory where one person is constantly getting shouted at and treated differently.

Similar to Follower 13’s experience of observing leader anger, Follower 15 (Sergeant) reported that he ‘[lost] a bit of respect’ for leaders who expressed anger when it was on a regular basis: ‘You lose a bit of respect for that person, because its unnecessary, you’re just like, you’re always like this, so we’re not going to pay as much attention to it.’

Follower 15 (Sergeant) also referred to the leader as a ‘*banger*’ (a personal insult). Such an insult suggests an arousal of negative follower emotion as well as reduced liking and respect for the leader, both of which are characteristic of an affective reaction. Therefore, this follower responded with both a negative affective reaction and a negative inference, which converged to have the overall outcome that the observer was more likely to ignore the angry individual in future.

### Asymmetrical outcomes of leader anger expression

There were many examples from followers of when leader expressions of anger resulted in the follower reflecting on their behavior, and subsequently the soldiers changed their behavior in an attempt to rectify the problem. As would be expected, these asymmetrical outcomes were strongly linked to the perceived appropriateness of the anger expression. Where the follower inferred that the anger was justified and fair, and its manner of expression was proportionate to the event causing it, they took notice and this provided them with a motivation to resolve that anger by changing their behavior. When asking Follower 19 (Sergeant) if throughout his career a leader had been angry toward him, he replied:

Yeah . . . I was smoking in Canada in a warrior . . . and I drove off with a [cigarette] in my mouth, and the company commander went apeshit. He got really angry on the radio. He said ‘get that fucking cigarette out of your mouth now’ [Laughs]. And I did . . . I did it because I realized I had forgotten where I was and forgotten what I was doing. He was right, and I was kind of shocked that I’d done it . . . I felt like a bit of an idiot . . . I sorted it immediately and nothing more was said.

Similarly, Follower 20 (Captain) reported being the target of leader anger, but, improved his performance as a result:

It’s usually from training, because I’ve done something wrong, or it’s usually when people are not performing to as high a standard as they could . . . I just accept it . . . it’s a good way of them displaying that they’re not happy with how you are performing.

Follower 20 (Captain) inferred that there was an issue with his performance, and so in this instance the leader anger was a useful tool in highlighting the problem and allowed him to address and rectify this problem.

As with Follower 20, Follower 8 (Lieutenant) described a time when his superior had been angry with him as he had not followed the appropriate protocol for reporting an incident involving one of his men:

I did get chewed at once by the adjutant, ’cos a soldier had a drunken mistake down in Wales; I was the officer in charge and then the chain of command found out about it because a brigadier complained to the commanding officer, so instead of me telling the adjutant, and him telling the commanding officer, it came all the way round, and they didn’t like finding out about things that way . . . there were a few expletives flying down the phone . . . but it was more like, ok, what is the situation, how can I get it sorted . . . they were just pissed off that I hadn’t told them.

From this narrative, Follower 8 (Lieutenant) seems to accept the anger expression as a result of his error, and subsequently made attempts to rectify his behavior. Clearly, leader expressions of anger are often accepted in line with pervading display norms. Furthermore, it is regarded as a constructive management tool in certain situations to draw urgent attention to followers in relation to process, performance or behavior issues. If the follower sees that this issue is attributable to them, then they accept the anger as a reasonable response (i.e. below the impropriety threshold).

Asymmetrical outcomes of leader anger would often also arise when anger was used in a controlled manner in combat and training situations. In these situations, the expression of anger resulted in the commanders’ orders being met swiftly and effectively, but also it ensured that soldiers would go and do what was asked of them (which in a threatening environment is likely to be in their best interests). Furthermore, anger expressions were often cited as having desirable consequences in training situations, as there was a need to emulate the sort of aggressive responses required in combat. Follower 7 (Captain) illustrates this:

If you’re on exercise and you’re doing a platoon attack . . . you’re rehearsing for a reality where if people do things incorrectly people will end up getting killed. That can result in a lot of screaming and a lot of shouting; you don’t want to be quietly tapping people on the shoulder and saying, come on, you could possibly do this a bit better. You need to get the point across immediately.

The reactions elicited in these high-pressure situations were affective in nature, as reportedly they were without any time for high levels of information processing and subsequent inferences. For example, Follower 15 (Sergeant) explained that:

. . . it’s key people like the Company Sergeant Major, if they come out and shout . . . it’s part of, you know, it needs to happen now, it’s an order. You’re in that mindset of needing to do the thing now, not when you feel like it.

Reactions to leader anger in these situations tended to be immediate affective responses, with Follower 7 (Captain) arguing that anger could be ‘*a very good tool to inject urgency into the situation*’ (affective reaction as emotional contagion, as mentioned earlier). In the same vein, Follower 20 (Captain) claimed that in his experience, leader expressions of anger constituted a ‘*matter of urgency that someone needs to do something, and that tends to work*’.

Interestingly, in very specialized contexts, such as certain training environments, inducing affective reactions in soldiers is a requirement for being able to deal with arduous situations:

I do remember doing the bayonet assault course. You’re being asked to put a bayonet on a rifle, and . . . alright at the time it’s into a sandbag, but you’re effectively sticking a foot and a half of steel into someone’s ribcage . . . they’re chanting kill, kill, kill . . . it’s exhausting because the instructors get you really angry and aggressive. It demonstrates the physical requirements of aggression needed for the job.

The affective reactions of soldiers here are mirrored by the leaders own expressions of aggression and anger, and subsequently this helps the soldiers achieve the aim of the exercise. However, this is not anger directed toward the leader who expressed the anger, it is directed toward the ‘enemy’ by way of mirroring the leader’s anger and becomes a useful form of emotional contagion.

## Discussion

This research project was guided by the question: *what conditions contribute to the expression of anger that crosses the impropriety threshold being seen as productive or counterproductive by observers or targets of that anger?* The data that emerged from our study led us to offer several important theoretical contributions. The first thing to note is that, in examining dynamic processes at work, simple models do not always capture the complexities that emerge at work. This seems particularly relevant in the context of display norms, as mentioned before. While we have outlined both evidence of negative outcomes of anger expressions (e.g. Lewis, 2000) and previous research regarding the positive outcomes of anger (e.g. Stickney and Geddes, 2014), our data revealed that both positive and negative outcomes emerged simultaneously. One factor influencing this is the paradoxical attitude toward expressions of anger, which reveal that, on the one hand, certain display rules are enforced to combat perceptions of a bullying culture (see account of Follower 12, Captain). On the other hand, however, there are certain characteristics of the situation and task at hand that seem to require the presence of anger to resolve these (see account of Follower 7, Captain). This tension, we suggest, exerts a constant pull and push on the location of the impropriety threshold. It is not static or fixed, but subject to continuous negotiation among those who express anger and the observers or targets of that expression. As our analysis suggests, one relevant factor or condition in that negotiation is the nature of task, especially when urgency had to be instilled into the situation. We note that, in the context of the DTM, the nature of the task as such is not explicitly mentioned as a factor prompting the crossing of the impropriety threshold.

A further issue arising from the data was the negative connotations followers associated with anger when it was either a consistent response or the only response from leaders. Several of the followers identified this as an issue inferring that a limited range of emotional expressions (i.e. only expressing anger) or expressing anger at a level that was inappropriate to the situation at hand resulted in the follower losing respect or liking for that leader (i.e. an effect beyond the impropriety threshold). This is partly consistent with prior research showing that the effects of anger diminish if it is constantly displayed (Lindebaum and Fielden, 2011). Certainly, there were examples in the data that supported this conclusion. Based on our data, we note that the impropriety threshold is not just about intensity of expression, but also about the consistency of the anger response. It appears that the impropriety threshold becomes reduced for observers when they perceive constant expressions of anger from leaders.

That said, however, we also found that there were times when expressing anger beyond the impropriety threshold was accepted as reasonable. For instance, Follower 19’s (Sergeant) recounting of one of his commanding officers going ‘apeshit’ and swearing at him. This would be considered in most workplaces (and in the military) as exceeding the impropriety threshold. In this case, however, the Follower found this response to be justified and accepted the anger expression without developing negative connotations, despite the breach of the impropriety threshold.

Our data also support research showing that expressions of anger result in observers or targets seeking information to resolve situations. In line with the EASI model (Van Kleef, 2014), we found that anger expressions resulted in followers reflecting on their own behavior to assist them in making sense of an anger expression (see also Van Kleef et al., 2009: for an example of enhanced follower performance as a result of leader anger expressions). We further note that affective reactions also produced asymmetrical outcomes by virtue of the nature of task at hand (i.e. instilling urgency in the situation), as already discussed above in the context of the DTM. In these instances, affective reactions took the form of emotional contagion, which transmitted leader anger and aggression to the followers.

## Practical implications

There are several broader practical implications we wish to draw attention to as well. First, the army seems to be explicit in terms of establishing expectations of behavior and associated display rules to address past and current accusations of leader bullying (Townsend, 2014). Our data, however, reveal a very real difference between display rules and display norms. Although there are explicit standards of acceptable behavior, our data reveal many implicit ‘norms’ that, together, dictate when a leader’s expression of anger is appropriate or not as perceived by observers and/or targets. Often, the accepted norm (freedom to express anger that exceeds the impropriety threshold at time) is not in line with organizational display rules. Some of this explains the apparent paradoxical attitude toward anger held by the military, as this and prior studies suggest (Linkh and Sonnek, 2003). We suggest that this is related to the special context within which all military organizations operate. However, these paradoxical tendencies can inhibit the very learning process at the heart of display norms. As Johnson et al. (2000) argue, display norms in organizations tend to be understood through a gradual learning process. Applied to our study, that gradual learning process is likely to be hampered vis-a-vis the paradoxical explicit and implicit information that both leaders and followers face. As our data have shown, anger can be quintessentially important vis-a-vis the task context of military (i.e. high-pressure combat situations), albeit some soldiers believed that the army has moved toward a much softer approach to training soldiers to avoid accusations of bullying. This would seem to suggest that the army places higher emphasis on emotional self-regulation by soldiers, and display rules are the conduit through which this expectation is communicated.

While there is still an inherent assumption and treatment of anger as an undesirable emotion in the military, the nature of some tasks to be performed in the context of the military nevertheless seems to necessitate it or in some cases produce it. The implications of this paradoxical situation, therefore, can be profound for military personnel, especially in terms of how they are socialized into, and trained for, the job. Our data indicate that there is a difference between display rules established by the organization in this context, the display norms required to achieve desired performance outcomes, the standards of behavior expected, and the preparation of soldiers for engaging in operational circumstances where extreme emotions are expected to be experienced. Soldiers need to be assisted in negotiating this dynamic process and a clear distinction provided for them between occasional anger utilized to enhance their training and performance and persistent angry responses, which may be interpreted as bullying.

Second, while remaining cautious not to generalize excessively from our data, results of this study may have relevance for employees in other organizational contexts, such as those who work in crisis situations (e.g. in the emergency services or police) or in organizations where significant and urgent change or action is required. Our study showcases anger as a particularly useful leadership tool in high-pressure situations where the safety or well-being of individuals or groups is paramount, owing to its potential to inject urgency and elicit immediate affective reactions and subsequent desirable behavioral responses in followers to meet the requirements of the situation.

Lastly, as observers of leader anger expression may react differently to those who are the target of the anger, leaders should be aware of the need to manage reactions to their anger in *both* observers and targets. Therefore, we suggest that leaders should be able to explain the reasons for their anger (at least post-hoc the event) not only to the target in order for them to make behavioral changes, but also to observers, whose reactions can be equally important to outcomes, even though they may not be directly involved. Being able to provide such explanations under conditions of ambiguity (e.g. the paradoxical situation in the military that anger expressions are both discouraged and encouraged) can help prevent attributional errors on the part of targets and observers (Geddes and Callister, 2007), and positively influence future leader–follower interactions (Dasborough and Ashkanasy, 2002). Indeed, this should be the other side of the EASI equation; that is, an angry person being able to explain their anger as well as the target seeking information about the anger.

## Limitations and future research

The method used in this study has inherent limitations, but the richness of the data analysis helps counterbalanced this. Qualitative data normally do not permit the testing of theories (Creswell, 1994). While it is crucial to recognize this limitation, it is worth-highlighting that the strength of qualitative research lies in the potential to refine our understanding (as is the case in this study) of existing theories (Pratt, 2009). Another potential limitation of the in-depth interview is that of interviewer bias (Antonakis et al., 2004). In order to limit researcher bias as an influencing factor in the interpretation of findings, we have followed clear guidelines for phenomenological research (Hycner, 1985) and have explained how this study complies with the ‘quality’ criteria for qualitative research as outlined by Lincoln and Guba (1985). Further to this, in line with all qualitative research (Kincheloe and Tobin, 2009), the phenomenological interview accepts the inability to generalize, and makes no such claims of generalizability or context-free results in this study (Ardley, 2011). With regards to the sample used, the study only included followers from one infantry regiment based in England and we acknowledge that no female respondents were interviewed. As such, findings may not be representative of the military at large, albeit one needs to bear in mind that – in phenomenological studies – larger samples do not necessarily yield *more* data (Sanders, 1982). Rather, we were concerned with providing ‘an account which allows for [a] depth of understanding, rather than one which unnecessarily generalizes the outcomes’ (Quinn, 2009: 258).

While this study has provided some theoretically and practically useful findings, there remain important areas for future research. First, we see the potential for future research to examine this phenomenon in other organizations and to expand the sample to include females. Future research would also benefit from considering the short- versus long-term outcomes of leader anger expressions. Our data revealed that constant displays of anger were related to an apparent lowering of the impropriety threshold. That is, in the long term, observers and targets dislike the angry individual, and will tolerate their anger less and will try to avoid future interactions (consistent with muted anger – see Geddes and Stickney, 2011). We can see that the exposure to constant anger might result in negative health consequences as a result of being exposed to leader anger over time (see Lawrence et al., 2011). This is one potential source for future research. There is also the opportunity to investigate the performance outcomes of experiencing constant anger. We can see that exposure to this kind of anger and the avoidance it generates could result in lower performance for observers and targets.

Although this has not been central to our original research question, we also note that display rules and norms in the military are perceived to be changing. As such, soldiers are left with conflicting ideas – owing to tensions between display rules and norms – of what is appropriate and what is not. While we know that these perceptions change over time (Stearns and Stearns, 1985), more research is required to understand *how* and *why* display norms change over time and how these norms are transferred within the workforce. For example, which factors (e.g. social, economic, environmental) induce display norms changes in organizations? How does this impact on how employees view display norms in their organization, both individually and collectively? How can we systematically study these changes over time, using which methods?

Finally, as already hinted at, exploring potential moderating effects of task-type in leadership studies is an important area for future research. Since the nature of tasks can vary significantly across industry and occupational contexts (compare, for example, the care of a nurse with the risk-taking of stock traders), it is plausible to suggest that this area offers significant potential for future research. As Johns (2006: 389) cautions, ‘context is likely responsible for one of the most vexing problems in the field: study-to-study’, adding that it often plays a larger role than individuals differences across research settings. More specifically, close appreciation of context (as it dictates the nature of tasks) can also aid in better understanding the missing linkages that can explain how activities of organizational members translate into organization-level outcomes (Goodman, 2000). Taken together, future research has the potential to progress both theoretically and empirically how leaders and followers interact emotionally to produce a variety of individual and organizational outcomes given a particular situation at hand within a certain moment in time.

## Conclusion

In sum, our study reveals interesting paradoxical attitudes toward expressions of anger in the context of leader–follower interactions in the military. While the military seeks to foster certain display rules to combat perceptions of a bullying culture, there are certain characteristics of the situation and task at hand that seem to require the presence of anger to resolve these. This helps explain why sometimes anger expression can yield symmetrical outcomes, whereas in other instances (either below or above the impropriety threshold) it leads to asymmetrical outcomes. Our study contributes to this ongoing debate by demonstrating that the impropriety threshold is not static or fixed, but subject to continuous negotiations among those who express anger and the observers or targets of that expression.

## Notes

1 For a full overview and description of the various ranks, please consult this website: http://www.army.mod.uk/structure/32321.aspx (accessed 19 February 2015).
